# Variations in the Impact of Gingerols’ Conversion to Shogaols on the Properties of Corn Starch with Different Amylose Contents

**DOI:** 10.3390/foods14010030

**Published:** 2024-12-26

**Authors:** Zhong Han, Wenhao Xiao, Yuhuan Geng, Diejia Cai, Xin’an Zeng, Wei Zhao, Wenhong Gao, Ji Ma

**Affiliations:** 1 School of Food Sciences and Engineering, South China University of Technology, Guangzhou 510640, China; 202410187146@mail.scut.edu.cn (W.X.); yhgeng@scut.edu.cn (Y.G.); 15521251679@163.com (D.C.); xazeng@scut.edu.cn (X.Z.); gaowh@scut.edu.cn (W.G.); 2 Guangdong Provincial Key Laboratory of Intelligent Food Manufacturing, Foshan University, Foshan 528225, China; 3 Overseas Expertise Introduction Center for Discipline Innovation of Food Nutrition and Human Health (111 Center), Guangzhou 510641, China; 4 China-Singapore International Joint Research Institute, Guangzhou 510700, China; 5 State Key Laboratory of Food Science and Technology, School of Food Science and Technology, Jiangnan University, Wuxi 214122, China; zhaow@jiangnan.edu.cn

**Keywords:** gingerols, shogaols, corn starch, amylose, interaction, structural properties

## Abstract

The polyphenol–starch complex has become a hot research topic since it is evident that this modification method can alter the physicochemical properties of starch as well as improve its nutritional value. This work aimed to evaluate the effect of ginger polyphenol gingerols (GNs) and shogaols (SNs) on the structure of starch with different amylose content (WCS, CS, G56, G80). Textural and rheological results indicated that GNs and SNs had more pronounced inhibitory retrogradation effects for relative low-level amylose starches (WCS and CS) compared to relative high-level amylose starches (G56 and G80). GNs and SNs improved the freeze–thaw stability of starch gels. FT-IR and XRD results revealed that GNs and SNs decreased the (short- and long-range) ordered structure of starches through a non-covalent interaction. Moreover, DSC results proved that the gelatinisation temperature of CS/G56/G80 significantly increased, and the enthalpy (ΔH) decreased by the incorporation of GNs and SNs. Overall, this in-depth study is beneficial in providing valuable pathways for starch–polyphenol interactions to improve the quality of starchy foods.

## 1. Introduction

Starch, composed of α-D-glucopyranose units, is a key food ingredient globally produced at over 60 million tons annually, among which 60% is used in food products for immediate consumption [1,2,3]. However, the utilisation of natural starches is limited due to their intrinsic drawbacks such as high viscosity, thermal instability and retrogradation properties during food processing [4]. Therefore, the development of suitable modification methods to overcome these shortcomings is crucial for the development of the starch industry. Polyphenols, bioactive substances that contain aromatic polyhydroxyl groups, have been extensively studied for their ability to modify the multiscale structure of starch during processing [5]. This modification imparts unique physicochemical properties, including retrogradation, textural and rheological properties [6,7].

Numerous studies have shown two main ways for the formation of starch–polyphenol complexes [8]: (1) the helical cavity is connected to the hydrophobic structure of polyphenols through hydrophobic interactions to form V-shaped complexes; (2) non-encapsulated complexes between the hydroxyl groups on polyphenols and starch molecules are formed through hydrogen bonding, hydrophobic interactions and Van der Waals forces [9]. Amoako and Awika [10] found that the hydrophobic flavonoid ring in anthocyanins can be embedded into the helical cavity of amylose, forming a V-shaped complex. The Van der Waals forces between hydrogen atoms on anthocyanins and on amylose glucose units, as well as the hydrogen bonding interactions between hydroxyl groups on anthocyanins and glucose, stabilise the structure of the complexes. He et al. [11] found that the decrease in apparent amylose content of corn starch is due to the interaction with longan seed polyphenols, which have a significant inhibitory effect on the digestion of corn starch. Li et al. [12] found that tea polyphenols decrease the gel strength and increase the viscosity and thermal stability of potato starch, due to the intramolecular hydrogen bond formed between tea polyphenols with modified starch molecules. However, starch–polyphenol non-encapsulated complexes usually do not contain V-shaped crystalline regions. This is possibly due to the large size of the polyphenols, the small helical space of the straight-chain starch and the insufficient hydrophobicity of the polyphenols that prevented the polyphenols from entering into the helical cavities of the starch. In addition, most of the polyphenols were bound to the starch by hydrogen bonding [13]. Karunaratne and Zhu [14] found that no V-type encapsulated complexes were formed between ferulic acid and corn starch, but the properties of corn starch such as swelling, pasting, retrogradation and gel structure were affected by ferulic acid through forces such as hydrogen bonding and hydrophobic interactions. Wei et al. [15] found that gallic acid, tannic acid and epigallocatechin gallate can spontaneously interact with amylose in a non-covalent form and that the higher the number of o–benzene–three–phenol, the tighter the intermolecular agglomeration, and the higher the dense structure and thermal stability of the complex. The pasting, gelation and ageing behaviour of starch can be affected by polyphenols by changing the spatial structure of starch. Wang et al. [16] found that the brassinose moiety on rutin enhances the solubility and dispersion of rutin in a starch solution and promotes the aggregation of starch granules and the formation of more rigid gels compared to quercetin. The results of this research have demonstrated that polyphenols can have a dramatic effect on the physicochemical properties of starch; hence, the aim of expanding the range of starch applications can be achieved.

Ginger (GR, *Zingiber officinale Roscoe*) is one of the most valuable economic crops of Zingiberaceae, which is widely used as a spice and flavouring in foods, because of its distinctive odour and flavour [17]. It is reported that China has the largest GR cultivation area in the world, covering about 300,000 hectares [18]. GR exerts a variety of physiological benefits such as reducing sickness and acting as an antioxidant, antibacterial and anti-inflammatory and enhancing digestive function [19,20,21,22]. The significant medicinal activity and unique pungent taste of GR are mainly derived from the polyphenol substances, which mainly include gingerols (GNs) and shogaols (SNs) [23]. It was reported that thermally unstable GNs can be dehydrated and converted to SNs during processing such as roasting, drying and carbonisation [24]. Both GNs and SNs have significant anti-inflammatory, hypoglycaemic, antioxidant and antidiabetic pharmacological activities [25]. In addition, as a traditional Chinese food, GR–starch soft candy is a functional food with potential health-promoting effects. However, to the best of our knowledge, there are no relevant articles on the effect of GR polyphenols on the physicochemical properties of starch. In particular, is it likely that GNs converted to SNs could cause a significant difference in modified starch?

Therefore, the first aim of the present work was to investigate the interaction between GR polyphenols (GNs/SNs) and corn starch with different amylose content to provide a theoretical basis for the application of GR polyphenols in modifying different types of starch. Secondly, since GNs are susceptible to dehydration and conversion to SNs, a comparative study of GNs and SNs in starch modification would be beneficial in providing guidance for a ginger–starch product design.

## 2. Materials and Methods

### 2.1. Materials

Corn starch with 56.0% and 80.0% amylose were named G56 and G80, respectively. G56, G80, waxy corn starch (WCS, 1.9% amylose) and normal corn starch (CS, 29.2% amylose) were purchased from Shanghai Eirean Co., Ltd. (Shanghai, China). Ginger was obtained from the local supermarket (Meizhou, China). Standard reagents were purchased from Chroma-Biotechnology Co., Ltd. (Chengdu, China). All other chemicals and reagents used in the study were of analytical grade.

### 2.2. Extraction of GNs and SNs

The GR was peeled and sliced, then mixed with 70% (*v*/*v*) aqueous ethanol at 1:10 (*w*/*w*) and crushed under high-speed stirring for 2 min to obtain fresh GR suspension. Suspension was extracted by ultrasonication (300 W, 25 °C) for 30 min and filtration to obtain the extract. This ultrasonication step was repeated twice. The whole extraction was centrifuged at 2654× *g* for 15 min, and the supernatant was concentrated into a viscous liquid at 50 °C. The concentrate was placed in an ultra-low temperature refrigerator at −80 °C for more than 8 h. Then, the frozen concentrate was freeze-dried to obtain the lyophilised powder and grinding. The fine powdered GNs extraction was stored in a dry environment and protected from light.

As GNs are easily dehydrated under high temperature and acidic conditions to produce SNs, high-temperature and long-time pretreatment of freshly cut GR slices was carried out to simulate the GR processing. The crushed GR grains were packed into beakers with a sealing film to reduce the loss of volatile substances and treated in an autoclave at 180 °C for 2 h. The SNs were extracted using the same procedure as for GNs [24].

### 2.3. Determination of GNs and SNs in Extract

The GR was peeled and sliced, then mixed with 70% (*v*/*v*) aqueous ethanol at 1:10 (*w*/*w*) and crushed under high speed. The standard solutions of 6-gingerols (6-G), 8-gingerols (8-G), 10-gingerols (10-G), 6-shogaols (6-S), 8-shogaols (8-S), 10-shogaols (10-S) (named for the number of carbon atoms in the alkane main chain) with different concentrations were used to obtain the standard curves for high-performance liquid chromatography (HPLC) (Hitachi L-8900, Hitachi Ltd., Tokyo, Japan). The determination of GNs was performed according to the method of Zhong et al. [26], and the column was an Agilent AQ-C18 column (4.6 mm × 250 mm, 5 μm) with 0.2% acetic acid in water (A) and acetonitrile (B) as mobile phases. The detection wavelength was 280 nm, and the column temperature was 25 °C. The injection volume was 20 μL, and the gradient elution condition was as follows: 0–45% B for 0–1 min, 45–65% B for 1–13 min, 65–80% B for 13–19 min and 80% B for 19–30 min at a flow rate of 1.0 mL/min. GNs and SNs extractions were dissolved by the methanol, then they were filtered through a 0.22 μm organic-phase membrane and loaded into liquid-phase vials for measurement. The contents of 6-G, 8-G, 10-G, 6-S, 8-S and 10-S in GNs and SNs were determined using HPLC as well.

### 2.4. Preparation of Starch–Polyphenol Gels and Freeze-Dried Powder

The GR was peeled and sliced. The starch (WCS, CS, G56, G80)–polyphenol (GNs/SNs) complexes were prepared according to the previous method with slight modifications [27]. In summary, 2 g of starch was added to 18 mL of distilled water. Concurrently, GNs and SNs (10% *w*/*w* relative to the dry starch basis) were dissolved in a 2 mL 80% ethanol solution. Then, the two solutions were mixed and stirred until homogeneity. Subsequently, the mixture was heated at 95 °C for 30 min and stirred to ensure homogeneity, after which it was poured into a cylindrical glass mould with a diameter and height of 1.5 cm. The samples were then transferred to a 4 °C refrigerator for 24 h to allow for retrogradation and gel formation (G56 and G80 were mixed with GNs/SNs in an oil bath at 150 °C for 30 min, while the other preparation steps above remained the same). The retrograded starch–GNs/SNs gels were submitted to textural and rheological analyses. Afterwards, the complex gels were placed into an ultra-low temperature refrigerator at −80 °C for over 8 h. Then, they were freeze-dried to obtain the lyophilised powder, which was ground and filtered (100-mesh sieve) to obtain the powder for freeze–thaw stability and DSC analysis.

### 2.5. Freeze–Thaw Stability

The freeze-dried starch–GNs/SNs complex (6.7%, *w*/*v*) was stirred and heated in boiling water for pasting and then cooled to 25 °C in a centrifuge tube [28]. After being stored at −18 °C for 24 h, the samples were thawed in a water bath at a temperature of 30 °C for 2 h. The starch emulsion was quickly placed into the cartridge of a filtered centrifuge tube and centrifuged at 2654× *g* for 10 min. The lower layer of water in the centrifuge tube was poured out, while the total mass of the sediment and the centrifuge tube were weighed to calculate the precipitation rate of starch according to the following formula:

$$Syneresls\left(\%\right)=\frac{Starch\;emulsion\;mass\left(g\right)-Precipitation\;mass\left(g\right)}{Starch\;emulsion\;mass\left(g\right)}\times 100$$

### 2.6. Gel Texture Properties

The retrograded starch–GNs/SNs gels with a diameter and height of 1.5 and 1.5 cm were prepared in Section 2.4. The TPA model was selected for the determination of gel texture properties using a physical texture measurement apparatus (TA-XT plus, Microsystems, Dresden, Sachsen, UK). The probe was a TA P/36R probe, in which the test speed (pre-test, test, post-test) was 1 mm/s. The trigger force was 0.05 N, and the compression strain was 30%. Two compressions with an interval of 5 s and 5–10 sets of measurements were performed.

### 2.7. Rheology Properties

The rheological properties of the samples fabricated in Section 2.4 were measured by a rotational rheometer (ARES-G2, TA Instruments Co., Ltd., New Castle, DE, USA) [8]. The geometry used was a parallel plate with a diameter of 30 mm and a gap of 1 mm. The samples were determined by amplitude scanning at 25 °C with an angular frequency range of 0.1–10 Hz (the strain was 1% within the linear viscoelasticity region). The trends of storage modulus (G′), loss modulus (G″) and tanδ (G″/G′) with respect to the angular frequency were recorded.

### 2.8. Particle Size

To investigate the effects of GNs and SNs on the particle size distribution of starch (WCS, CS, G56 and G80), GNs and SNs (at a concentration of 10% based on starch) were added to an aqueous solution of starch (with a concentration of 10%, *w*/*v*) and stirred at 30 °C for 2 h. Subsequently, the starch emulsion was centrifuged at 2654× *g* for 10 min and the precipitate was washed with 50% anhydrous ethanol and distilled water 3 times, respectively. The resulting powder was then lyophilised, ground through a 100-mesh sieve, and subjected to tests. The particle size variation of the samples was characterised using a laser particle sizer (Malvern Instruments, Worcestershire, UK). Briefly, the samples were dispersed in distilled water to a concentration of 10 mg/mL, and to obtain the appropriate shading factor (10–20%).

### 2.9. Scanning Electron Microscopy (SEM)

To investigate the effects of GNs and SNs on the microstructural property of WCS, CS, G56 and G80, the samples prepared in Section 2.8 were observed under a scanning electron microscope at 10 kV and 11,000× magnification (EVO18, Zeiss Co., Ltd., Oberkochen, Germany) [29].

### 2.10. X-Ray Diffraction (XRD)

X-ray diffraction patterns of samples prepared in Section 2.8 were used to characterise the crystal morphology and relative crystallinity at 40 kV and 40 mA (D8 ADVANCE, Bruker Co., Ltd., Karlsruhe, Germany) [30]. The diffraction angle ranged from 5° to 35° at a scanning rate of 1°/min, and the relative crystallinity (R) was calculated using MD Jade 6.5 software (Material Data Corporation, Livermore, CA, USA).

### 2.11. Fourier Transform-Infrared Spectroscopy (FT-IR)

FT-IR was used to characterise the effect of GNs/SNs on the functional groups of starch prepared in Section 2.8. The absorption spectra of samples were scanned by ATR between the 4000–650 cm^−1^ wavenumber range at a resolution of 4 cm^−1^ (Spectrum 3, PerkinElmer Co., Ltd., Waltham, MA, USA). The degree of the short-range ordered structure (1047 cm^−1^/1022 cm^−1^) was calculated by OMNIC software, v 9.1 (Thermo Fisher Scientific Corporation, Madison, WI, USA) [31].

### 2.12. Differential Scanning Calorimetry (DSC)

The thermodynamic properties of the starch–GNs/SNs complexes powder prepared in Section 2.4 were determined by differential scanning calorimetry (DSC 8000, Perking Elmer, Waltham, MA, USA) [32]. A mixture of starch–GNs/SNs (5 mg) and deionized water (15 μL) was sealed in an aluminium pan and equilibrated overnight at 25 °C and then scanned from 30 to 90 °C at 10 °C/min.

### 2.13. Statistical Analysis

All the experiments were performed at least in triplicates. Data were analysed by Duncan’s multiple-range test (*p* < 0.05) using the SPSS 17.0 statistical software program (SPSS Incorporated, Chicago, IL, USA).

## 3. Results and Discussion

### 3.1. Determination of GNs/SNs Components

The type and content of polyphenol in the GNs and SNs extractions were determined by high-performance liquid chromatography. 6-G, 8-G, 10-G and 6-S were the mainly detected extracts, among which 6-G accounted for the largest proportion of about 70.8% in the GNs (Table 1).

In contrast, 6-S was very minimal. Thus, the most characteristic bioactive substances in fresh GR are GNs, which is similar to the results of An et al. [33]. 6-S, 8-S and 10-S were mainly detected in the SNs extract, among which 6-S was the most abundant, accounting for 74.05%. There were no GNs components (6-G, 8-G, 10-G) detected in the SN extracts, suggesting that the GNs were entirely converted to SNs in response to heating. Ghasemzadeh et al. [34] found that increased temperature and prolonged, hot, air-drying treatment time resulted in a significant decrease in the GNs content and a corresponding increase in shogaol content. These results demonstrated the thermal instability of GNs and the fact that thermal treatment is capable of converting GNs into SNs.

### 3.2. Freeze–Thaw Stability

**Figure 1:**
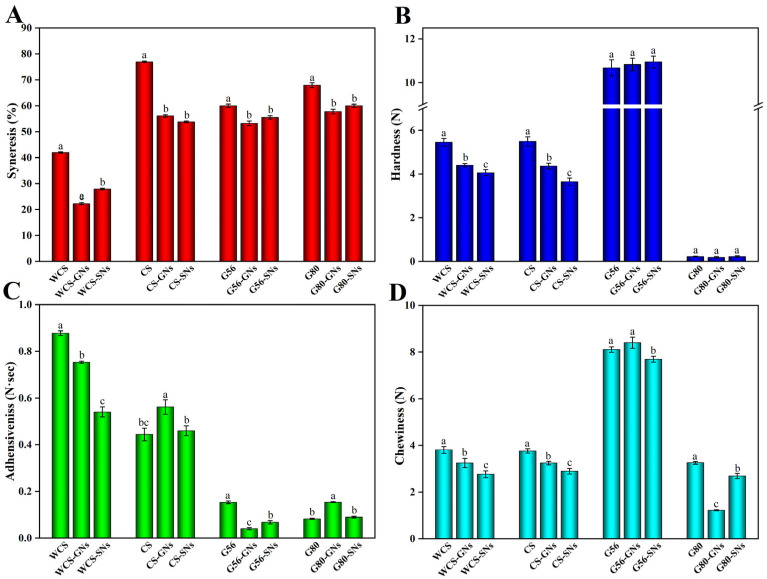
Freeze–thaw stability (**A**), hardness (**B**), adhesiveness (**C**) and chewiness (**D**) of starch–polyphenol complexes. a–c: different lowercase letters in the same group are significantly different (*p* < 0.05).

As shown in Figure 1A, all starch–GNs/SNs complex gels exhibited a decreased syneresis rate, indicating that GNs and SNs promoted the water-holding properties of starch gels with better freeze–thaw stability. In particular, GNs and SNs affected the freeze–thaw stability of WCS and CS remarkably. For example, the syneresis rate of WCS–GNs and CS–GNs were reduced from 42% and 77% to 20% and 58%, respectively. 

The syneresis rate was notably reduced by about 20%. Compared to WCS, as the content of amylose increased, the freeze–thaw stability of CS, G56 and G80 became worse, which might be due to the fact that the molecular weight of amylose is smaller than amylopectin, resulting in weaker hydrogen bonding with water molecules [4]. It has been reported that the freeze–thaw stability of hydrogels was related to the molecular weight of the gel-forming molecules where higher molecular weights are attributed to lower syneresis [35]. The binding of GNs/SNs with neighbouring water molecules makes it difficult for water molecules to be released from the network gel structure [36]. In addition, the hydroxyl groups of GNs/SNs may affect the interaction between starch and water molecules such as hydrogen bonding and Van der Waals forces [37]. Therefore, GNs and SNs can ameliorate the undesirable physical changes that may occur during the freezing and thawing of starch-based foods, thereby retarding the retrogradation of starchy foods.

### 3.3. Gel Textural Properties

Gel hardness is an important indicator to evaluate food texture, and it is highly related to retrogradation of starch [38]. As shown in Figure 1B, the extremely weak hardness of G80 is due to the excessive content of amylose, which prevents the formation of a stable gel network structure. The hardness and chewiness of G56 gels were more significant, and the GNs and SNs had little effect on their gel properties. However, GNs and SNs can notably reduce the hardness and chewability of WCS and CS, and they serve as inhibitors of starch retrogradation. In addition, SNs showed stronger inhibitory effects than GNs. These results are consistent with previous studies reporting that starch–phenolic complexes exhibited an inhibition of starch degradation successfully [39,40]. These different results occurred in G56 and WCS/CS probably due to variations in the content of amylose. Polyphenols are more likely to interact with amylose thus affecting starch retrogradation, and as the relative content of amylose is lower in WCS/CS, it generates a greater impact as a result of interaction with the same quantity of polyphenols. It indicated that GNs and SNs had a more noticeable inhibitory retrogradation effect on starch with low-level amylose (WCS and CS). The adhesiveness of the WCS groups was higher and exhibited a stronger adhesive behaviour (Figure 1C). This is attributed to the fact that amylopectin with high hydroxyl density provides an excellent adhesive force [41].

### 3.4. Rheology Properties Analysis

**Figure 2:**
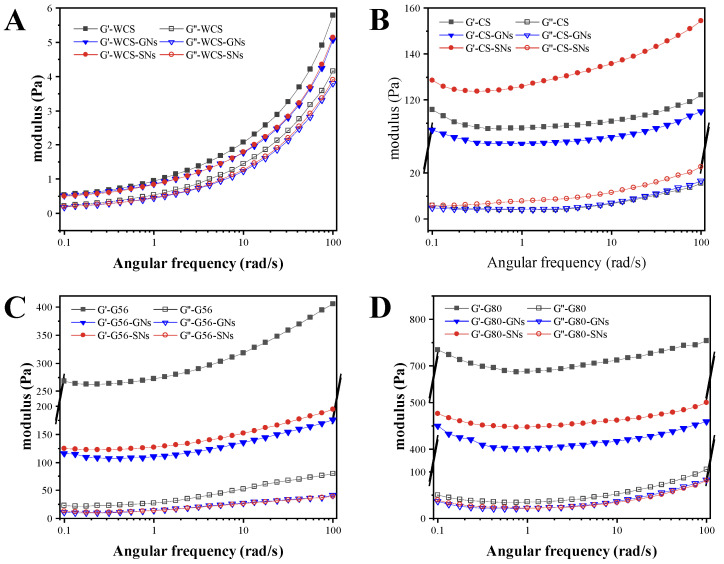
Rheology properties of GNs/SNs with WCS (**A**), CS (**B**), G56 (**C**) and G80 (**D**) complexes.

The viscoelasticity of starch–GNs/SNs complexes is shown in Figure 2. Both G′ and G″ of the WCS and WCS-GNs/SNs complexes were very small, with G′ being slightly larger than G″, suggesting that the amylopectin content of the mainly composed WCS has a very weak gel behaviour (Figure 2A). The addition of GNs and SNs also had little effect on G′ and G″, suggesting that interactions between GNs/SNs and highly branched amylopectin hardly affect the properties of the gel network structure. Chai et al. [42] confirmed that tea polyphenols tend to interact with amylose rather than amylopectin. As shown in Figure 2B, the addition of GNs decreased the G′ of the CS gels, while the effect on G″ was not significant, suggesting that the GNs mainly affected the elastic properties of the gels, leading to a decrease in the elasticity of the gel network structure. The addition of SNs produced a significant increasing effect on both G′ and G″ of the CS gels, which suggested that the SNs significantly enhanced the viscoelasticity of the starch gel. As shown in Figure 2C,D, the G′ and G″ of G56/G80 significantly decreased by GNs and SNs, suggesting that maybe the interactions between GNs/SNs and amylose can promote structural disintegration.

### 3.5. Particle Size Distribution Analysis

**Figure 3:**
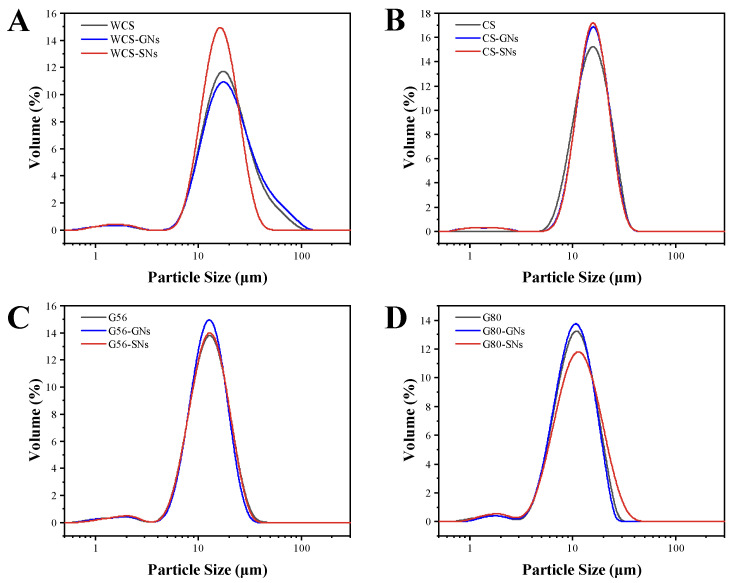
Effect of GNs and SNs on the particle size distribution of WCS (**A**), CS (**B**), G56 (**C**), G80 (**D**).

Figure 3 and Table 2 show the effect of a 10% addition of GNs/SNs extracts on the particle size distribution of WCS, CS, G56 and G80. There were two peaks in the particle size distribution of WCS and its complexes, 18.22 μm and 1.54 μm, which were similar to the previous study [43]. 

The particle size of GNs–WCS was increased, while the particle size of SNs–WCS was reduced. Liu et al. [44] found that the larger molecular tannins and proanthocyanidins constituted a starch–polyphenol complex with a larger particle size, whereas the smaller molecules of quercetin constituted a starch–polyphenol complex with a smaller particle size. The insignificant change in D _[4,3]_ and the decrease in D _[3,2]_ of the CS complexes indicated that GNs/SNs do not have a significant effect on the bulk particle size of the CS, but GNs/SNs can disrupt the surface structure of the CS to expose a larger specific surface area. GNs/SNs may enter into the hydrophobic inner cavity of a straight-chain starch due to its hydrophobicity and is unlikely to contribute to its particle size increase. There was no significant change in the position of the main peak and particle size of the G56 complex. The particle size of GNs–G80 was slightly smaller, and the particle size of G80–SNs was larger, which indicated that SNs were able to disrupt and expose more surface to link starch fragments.

### 3.6. Scanning Electron Microscopy (SEM) Analysis

**Figure 4:**
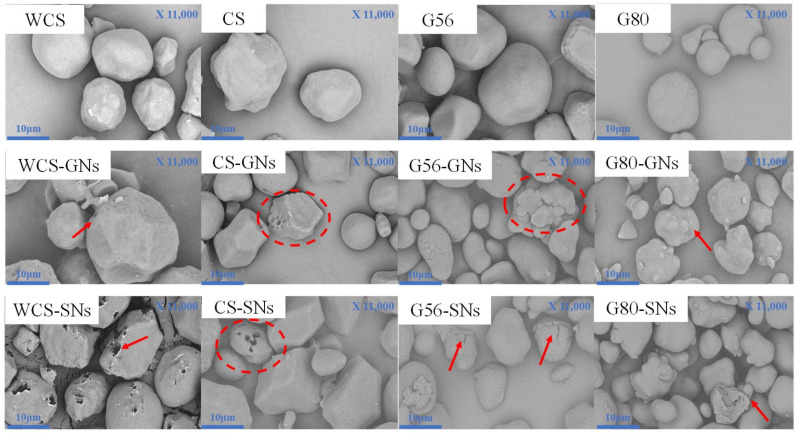
Effect of GNs and SNs on the microstructure of WCS, CS, G56 and G80.

The effect of GNs and SNs on the apparent morphology of the starch granules was performed by SEM. As shown in Figure 4, most of the native WCS and CS were spherical particles with smooth surfaces and structural integrity, while others appeared to be polygonal prisms. The surface of WCS and CS granules showed different degrees of disruption. Among them, the SNs–WCS granules were disrupted more significantly, which had more (and larger) pores and a larger degree of surface collapse. It may be that amylopectin in WCS was more easily exposed though the disrupting effect on the surface structure of starch granules by SNs. The surface structure of CS was little affected by the GNs and SNs, with only a few areas showing minor depressions and roughness. This may be due to the dense structure of CS and the strong interaction force between straight-chain starch and branched-chain starch, which made the microstructure of CS less susceptible to interference by external factors. Castanha et al. [45] reported that even under strong external forces such as ozone and ultrasound, it was difficult to significantly change the apparent structure of ordinary corn starch, and only minor pore formation was observed.

The surface of G56 particles was smooth, and the particle size was relatively large. A large number of spherical particles were attached to the surface of the G56 particles in the GNs–CS group. This might be due to the GNs’ function as a bridge connecting the short amylose starch and the amorphous-zone starch in the aggregation state to form ordered aggregates of larger size and denser structure, whereas the surface porosity of the G56 starch increased, and the surface cracks were obvious after interaction with SNs. It was speculated that during the heat-absorbing pasting process, the SNs–G56 starch chain-spacing and space steric hindrance became larger, forming more loosely structured crystalline regions. Lopez-Rubio et al. [46] found that acylation modification of high amylose chain starches by shorter-chain fatty acids (e.g., butyric acid) resulted in larger voids and structural changes. It is attributed to the fact that the short-chain fatty acids are perpendicular to each other and continue to extend with the amylose starch, leading to greater disruption in the morphology and structure of the granules. The size of G80 starch granules was small and heterogeneous. After the GNs and SNs treatment, the shape of G80 became irregular with many small spherical particles attached to the surface. Especially in the SNs-treated group, a few G80 starch surfaces showed a severe collapse, which indicated the existence of non-covalent bonds such as hydrogen bonding and hydrophobic interactions of GNs/SNs with G80, but such interactions are mainly short-chained amylose starch. Zhao et al. [47] found that with the addition of tea polyphenols, the bumps on the starch surface became larger and gradually integrated with the starch granules. It is possible that the starch granules form a stronger interaction force with the tea polyphenols [48].

By analysing the effects of GNs and SNs on the apparent morphology of corn starch with different amylose content, it can be seen that the degree of destruction of the surface structure of the starch granules by GNs and SNs gradually decreased with the increase of amylose content. The surface of the waxy corn starch had deeper and more numerous pores, while the structure of high amylose corn starch was more irregular, partially destroying the lamellar structure of the starch. This may indicate that corn starch with a higher content of amylose has a denser crystalline layer, which makes it more difficult to disrupt.

### 3.7. Fourier Transform-Infrared Spectroscopy (FTIR) Analysis

**Figure 5:**
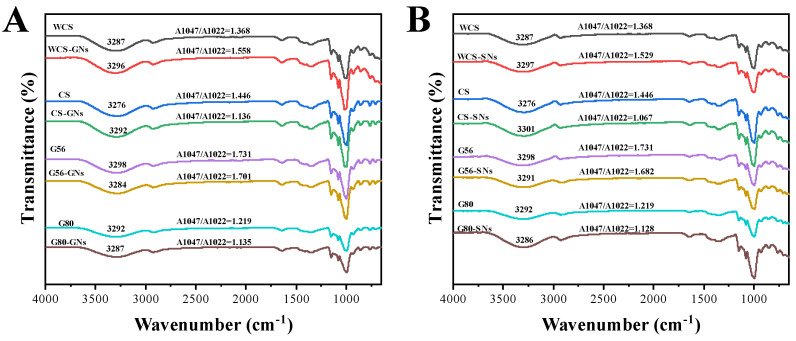
FTIR spectra of ginger polyphenols GNs (**A**) and SNs (**B**) complexed with starch.

FT-IR spectra were used to analyse the structural information and interaction between the starch and GNs/SNs. The peak at 1047 cm^−1^ represented the crystalline region of starch, which is proportional to the degree of the orderliness of the starch granules. The peak at 1022 cm^−1^ was an indication of amorphous region, and an increase in its peak indicated a more disordered state of the starch. The degree of the short-range ordered starch was generally reflected by the value of 1047 cm^−1^/1022 cm^−1^, with larger values implying a higher degree of ordered structure [49]. Figure 5A shows that compared to normal starch, no novel chemical bond and functional group were observed in the FT-IR spectra of the GNs/SNs–starch. This suggested that there was probably non-covalent interaction between starch and GNs/SNs, such as hydrogen bonds and hydrophobic interactions, etc. Figure 5A shows that the value of 1047 cm^−1^/1022 cm^−1^ increased in the GNs/SNs–WCS complexes, indicating that the short-range ordering of the GNs/SNs–WCS complexes increased. Hydrogen interactions between amylopectin and phenolic hydroxyl groups likely caused this effect. The amylopectin is able to further interact with itself, leading to the formation of more ordered, double-helical structures and enhancing the degree of intermolecular aggregation during gel ageing [50,51]. As shown in Figure 3, the 1047 cm^−1^/1022 cm^−1^ ratio of the GNs/SNs-CS/G56/G80 groups decreased, suggesting that the GNs/SNs led to poorer, short-range ordering and reduced the recrystallisation of starch in the CS/G80 retrogradation process [52].

### 3.8. X-Ray Diffraction (XRD) Analysis

**Figure 6:**
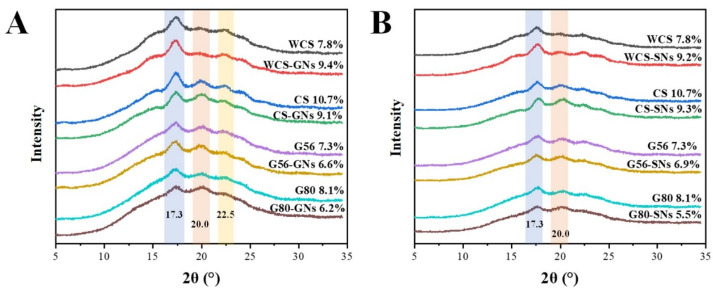
XRD patterns of GNs (**A**) and SNs (**B**) complexed with starch.

The XRD patterns of GNs/SNs–starch samples are shown in Figure 6. The results proved that the WCS and WCS–GNs/SNs exhibited a typical B crystalline shape after retrogradation, with a distinct diffraction peak at 17.3°. The crystalline shape of WCS was not changed by the incorporation of GNs and SNs, while the relative crystallinity increased. It suggested that starch retrogradation cannot be inhibited by GNs and SNs due to their weak binding capacity to amylopectin [53]. Others exhibited a typical B + V crystalline shape, with a distinct diffraction peak at 17.3° and 20.0°. The crystalline shape of starch was not changed by the incorporation of GNs and SNs. The relative crystallinity of the samples decreased due to the addition of GNs and SNs, which may be attributed to the fact that CS, G56 and G80 contain different proportions of amylose. The crystallisation of molecular aggregates in the amorphous region was inhibited through GNs and SNs into the amylose cavities to achieve a delayed starch retrogradation effect [54].

### 3.9. Thermodynamic Properties Analysis

**Figure 7:**
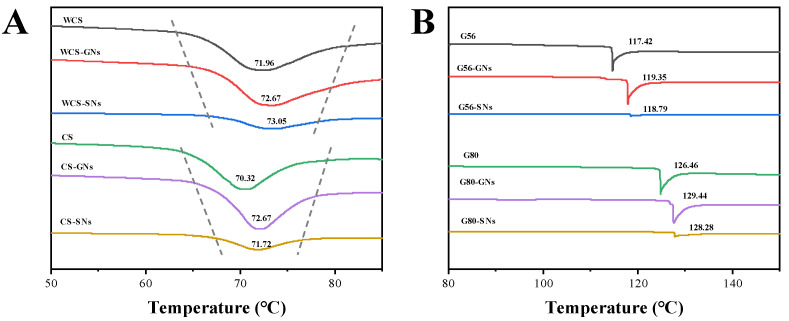
DSC curves of WCS and CS (**A**), G56 and G80 (**B**) with GNs/SNs.

The DSC curves of complexes are shown in Figure 7, and the results of the thermodynamic parameters Onset temperature (To), Peak temperature (Tp), Conclusion temperature (Tc) and Enthalpy (ΔH) are shown in Table 3. 

In the starch–GNs/SNs complexes, the Tp was elevated with the addition of GNs and SNs. The increase in ΔH of the WCS–GNs/SNs complex may be due to the fact that the amylopectin chains are more able to bind to the neighbouring side chains to form a dense structure under the action of the GNs/SNs, which requires higher temperatures and greater energies to unfold the double-helix structure and the crystalline regions [55]. The gelatinization temperature (Tc-To) of CS/G56/G80-GNs/SNs significantly increased, and the enthalpy (ΔH) decreased, suggesting that less energy was required to disrupt the double helix in complexes and that GNs/SNs can inhibit CS/G56/G80 starch retrogradation. Wang et al. [56] found that soy isoflavones significantly increased the gelatinization temperature and decreased the enthalpy of gelatinization of corn starch. It is possible that the hydration of the starch granules and the hydrogen bonding interactions between the starch chains are affected by the binding of the hydroxyl groups of the isoflavones to the starch granules, disrupting the ordered structure, leading to a reduction in the energy required to disrupt the double helix.

## 4. Conclusions

In summary, this work demonstrated that there was a significant effect of GNs/SNs on the rheological, textural, microstructural and the thermal stability of starch. GNs/SNs had different degrees of disruptive effects on the surface structure of WCS and CS but showed more remarkable effects on G56 and G80. GNs and SNs can inhibit the CS/G56/G80 retrogradation. SNs significantly enhanced the viscoelasticity of the starch–gel structure. GNs and SNs improved the water-holding properties of starch gels with better freeze–thaw stability. The hardness, adhesiveness and chewiness of the CS group were relatively moderate, while the addition of GNs and SNs decreased the hardness and chewability of CS. In a nutshell, this study contributes to increasing a better understanding on GR polyphenol GNs/SNs to modify starch, which is important to the design and regulation of starch-based products.

## Figures and Tables

**Table 1:** The content of gingerol and shogaol in the extracts.

	6-G (mg/g)	8-G (mg/g)	10-G (mg/g)	6-S (mg/g)	8-S (mg/g)	10-S (mg/g)	Total (mg/g)
GNs	0.6281 ± 0.0414	0.0893 ± 0.012	0.0964 ± 0.0018	0.0723 ± 0.0028	-	-	0.8861 ± 0.0302
SNs	-	-	-	0.7027 ± 0.0019	0.1507 ± 0.0023	0.0951 ± 0.0005	0.9485 ± 0.0923

Note: Data are expressed as the mean ± standard deviation (n = 3), “-” indicates not detected.

**Table 2:** Effect of GNs/SNs on the particle size distribution of starch.

	D _[4,3]_	D _[3,2]_	D10 (μm)	D50 (μm)	D90 (μm)
WCS	21.69 ± 0.51 b	13.44 ± 0.17 a	9.70 ± 0.12 a	18.22 ± 0.27 a	38.31 ± 1.46 b
WCS-GNs	23.43 ± 0.84 a	13.78 ± 0.25 a	9.70 ± 0.14 a	18.88 ± 0.42 a	43.70 ± 2.60 a
WCS-SNs	16.80 ± 0.07 c	12.14 ± 0.07 b	9.38 ± 0.02 b	15.92 ± 0.05 b	25.98 ± 0.13 c
CS	16.15 ± 0.07 a	14.2 ± 0.07 a	9.29 ± 0.07 b	15.32 ± 0.07 a	24.32 ± 0.08 b
CS-GNs	16.05 ± 0.08 a	11.92 ± 0.03 b	9.71 ± 0.03 a	15.49 ± 0.06 a	23.67 ± 0.19 b
CS-SNs	15.97 ± 0.05 a	11.88 ± 0.07 b	9.77 ± 0.02 a	15.43 ± 0.05 a	23.46 ± 0.09 a
G56	13.33 ± 0.03 a	9.79 ± 0.02 b	7.01 ± 0.01 a	12.51 ± 0.03 a	21.07 ± 0.06 a
G56-GNs	12.93 ± 0.03 b	9.78 ± 0.01 b	7.18 ± 0.03 a	12.33 ± 0.02 b	19.93 ± 0.13 b
G56-SNs	13.24 ± 0.06 a	9.97 ± 0.02 a	7.03 ± 0.01 a	12.49 ± 0.03 a	20.84 ± 0.10 b
G80	11.11 ± 0.06 b	8.51 ± 0.03 b	5.69 ± 0.02 a	10.43 ± 0.05 b	17.83 ± 0.11 b
G80-GNs	10.82 ± 0.03 c	8.65 ± 0.02 a	5.71 ± 0.01 a	10.24 ± 0.02 b	17.07 ± 0.08 b
G80-SNs	12.15 ± 0.09 a	8.75 ± 0.03 a	5.59 ± 0.01 b	11.03 ± 0.05 a	20.50 ± 0.19 a

Data are expressed as the mean ± standard deviation (n = 3). Values of means followed by different lowercase letters in the same column are significantly different (*p* < 0.05).

**Table 3:** Thermal properties of starch granules and their complexes with GNs/SNs.

Samples	Onset Temperature To (°C)	Peak Temperature Tp (°C)	Conclusion Temperature Tc (°C)	Enthalpy ΔH (J/g)
WCS	67.44 ± 0.54 e	71.96 ± 0.82 de	78.47 ± 0.41 d	11.55 ± 0.07 d
WCS-GNs	67.57 ± 0.50 e	72.67 ± 1.45 d	79.72 ± 0.54 d	12.41 ± 0.59 bc
WCS-SNs	67.34 ± 1.32 e	73.05 ± 0.55 d	79.92 ± 0.27 d	12.03 ± 0.10 c
CS	65.55 ± 0.30 f	70.31 ± 0.45 e	74.70 ± 0.23 f	12.10 ± 0.22 c
CS-GNs	67.58 ± 0.58 e	72.67 ± 0.36 d	76.57 ± 0.36 e	11.21 ± 0.10 d
CS-SNs	67.35 ± 0.49 e	71.72 ± 0.12 de	76.80 ± 0.17 e	10.55 ± 0.54 d
G56	117.32 ± 1.23 d	117.42 ± 1.11 c	119.33 ± 1.35 c	14.39 ± 1.52 b
G56-GNs	118.24 ± 0.53 d	119.35 ± 1.10 c	120.35 ± 1.25 c	14.21 ± 1.42 b
G56-SNs	119.23 ± 0.11 c	118.79 ± 2.67 c	120.07 ± 1.04 c	12.56 ± 0.96 c
G80	126.32 ± 1.35 b	126.46 ± 0.80 b	128.88 ± 1.84 b	18.42 ± 2.31 a
G80-GNs	129.32 ± 0.63 a	129.44 ± 0.45 a	131.67 ± 2.34 ab	14.54 ± 2.31 b
G80-SNs	128.18 ± 0.54 ab	128.28 ± 0.74 ab	130.06 ± 0.35 b	6.32 ± 0.86 e

Data are expressed as the mean ± standard deviation (n = 3). Values of means followed by different lowercase letters in the same column are significantly different (*p* < 0.05).

## Data Availability

The original contributions presented in the study are included in the article, further inquiries can be directed to the corresponding authors.
